# AI Model Based on Diaphragm Ultrasound to Improve the Predictive Performance of Invasive Mechanical Ventilation Weaning: Prospective Cohort Study

**DOI:** 10.2196/72482

**Published:** 2025-09-08

**Authors:** Feier Song, Huazhang Liu, Huan Ma, Xuanhui Chen, Shouhong Wang, Tiehe Qin, Huiying Liang, Daozheng Huang

**Affiliations:** 1 Department of Emergency and Intensive Care Unit, Guangdong Provincial People’s Hospital (Guangdong Academy of Medical Sciences), Southern Medical University, Guangzhou, 510080, China; 2 Medical Big Data Center, Guangdong Provincial People’s Hospital (Guangdong Academy of Medical Sciences), Southern Medical University, Number 106, Zhongshaner Road, Guangzhou, 510080, China, 86 13416404410; 3 Department of Cardiology, Guangdong Provincial People’s Hospital (Guangdong Academy of Medical Sciences), Southern Medical University, Guangdong Provincial Cardiovascular Institute, Guangzhou, 510080, China; 4 Department of Critical Care Medicine, Guangdong Provincial People’s Hospital (Guangdong Academy of Medical Sciences), Southern Medical University, Guangdong Provincial Geriatrics Institute, No. 106, Zhongshaner Rd, Guangzhou, 510080, China, 86 15920151904; 5 Office of Organ Procurement Organizations, Medical Department, Guangdong Provincial People’s Hospital (Guangdong Academy of Medical Sciences), Southern Medical University, Guangzhou, 510080, China

**Keywords:** deep learning, multimodal learning, point-of-care ultrasound, POCUS, critical care

## Abstract

**Background:**

Point-of-care ultrasonography has become a valuable tool for assessing diaphragmatic function in critically ill patients receiving invasive mechanical ventilation. However, conventional diaphragm ultrasound assessment remains highly operator-dependent and subjective. Previous research introduced automatic measurement of diaphragmatic excursion and velocity using 2D speckle-tracking technology.

**Objective:**

This study aimed to develop an artificial intelligence–multimodal learning framework to improve the prediction of weaning failure and guide individualized weaning strategies.

**Methods:**

This prospective study enrolled critically ill patients older than 18 years who received mechanical ventilation for more than 48 hours and were eligible for a spontaneous breathing trial in 2 intensive care units in Guangzhou, China. Before the spontaneous breathing trial, diaphragm ultrasound videos were collected using a standardized protocol, and automatic measurements of excursion and velocity were obtained. A total of 88 patients were included, with 50 successfully weaned and 38 experiencing weaning failure. Each patient record included 27 clinical and 6 diaphragmatic indicators, selected based on previous literature and phenotyping studies. Clinical variables were preprocessed using OneHotEncoder, normalization, and scaling. Ultrasound videos were interpolated to a uniform resolution of 224×224×96. Artificial intelligence–multimodal learning based on clinical characteristics, laboratory parameters, and diaphragm ultrasonic videos was established. Four experiments were conducted in an ablation setting to evaluate model performance using different combinations of input data: (1) diaphragmatic excursion only, (2) clinical and diaphragmatic indicators, (3) ultrasound videos only, and (4) all modalities combined (multimodal). Metrics for evaluation included classification accuracy, area under the receiver operating characteristic curve (AUC), average precision in the precision-recall curve, and calibration curve. Variable importance was assessed using SHAP (Shapley Additive Explanation) to interpret feature contributions and understand model predictions.

**Results:**

The multimodal co-learning model outperformed all single-modal approaches. The accuracy improved when predicted through diaphragm ultrasound video data using Video Vision Transformer (accuracy=0.8095, AUC=0.852), clinical or ultrasound indicators (accuracy=0.7381, AUC=0.746), and the multimodal co-learning (accuracy=0.8331, AUC=0.894). The proposed co-learning model achieved the highest score (average precision=0.91) among the 4 experiments. Furthermore, calibration curve analysis demonstrated that the proposed colearning model was well calibrated, as the curve was closest to the perfectly calibrated line.

**Conclusions:**

Combining ultrasound and clinical data for colearning improved the accuracy of the weaning outcome prediction. Multimodal learning based on automatic measurement of point-of-care ultrasonography and automated collection of objective clinical indicators greatly enhanced the practical operability and user-friendliness of the system. The proposed model offered promising potential for widespread clinical application in intensive care settings.

## Introduction

Invasive mechanical ventilation (MV) is a life-saving therapeutic strategy in critically ill patients, yet 20%‐40% of invasive MV patients endure difficult weaning to varying extents [1]. WEAN-SAFE study, a prospective cohort of 10,232 invasive MV patients in 481 intensive care units (ICU) among 50 countries, showed that the incidence of difficult weaning, prolonged weaning, and weaning failure reached up to 35.3% [2]. Diaphragm dysfunction is a major concern in critically ill patients due to its potential association with prolonged MV dependency [3,4], as the diaphragm plays a central role in respiratory mechanics.

The use of point-of-care ultrasonography (POCUS) to assess the diaphragm has seen rapid growth within the ICU because it is readily available, portable, noninvasive, visual, easily repeatable, and free of ionizing radiation [5]. Previous systematic reviews have examined the accuracy and usefulness of diaphragmatic ultrasound indicators in predicting weaning outcomes, mainly focusing on diaphragm thickening fraction (DTF), excursion, and rapid shallow breathing index [6-10]. Encouragingly, our serial research, published in January 2023, developed an automatic measurement of diaphragm excursion and velocity based on 2D speckle-tracking technology, showing that the mean diaphragm excursion and velocity had a high diagnostic value on the weaning outcomes [11].

In parallel, multimodal artificial intelligence (AI), or multimodal learning, enabling the AI/machine learning (ML) model to learn from and process multiple modes and types of data (image, text, audio, and video) rather than just one, is a rising trend and has the potential to reshape both the AI landscape and clinical research for its novel implications and benefits. By integrating AI into the evaluation process, clinicians can receive more accurate and consistent predictions, allowing for better decision-making. Furthermore, AI-guided prediction models can adapt to the individual characteristics of patients, offering personalized insights that improve the safety and efficacy of the weaning process [12,13].

To further extend our findings, this study aims to develop and validate a multimodal AI model that integrates clinical characteristics, laboratory parameters, and diaphragm ultrasound videos from the abovementioned cohort. This might provide multisystem and multiorgan morphological and functional status information, which helps predict weaning failure and guide the personalized, accurate weaning strategies in the ICU.

## Methods

### Study Design

Patients aged older than 18 years who received MV for more than 48 hours, suitable for a spontaneous breathing trial (SBT), were prospectively enrolled in ICUs within 2 hospitals in Guangzhou City, China. Ultrasound video and diaphragm excursion and velocity were acquired, followed by a standardized protocol using the TE7 Diagnostic Ultrasound System (C5-2 array probe, Shenzhen Mindray Biomedical) in a supine or semirecumbent position before SBT by a well-trained expert. Of the total 88 critically ill patients, 50 (57%) patients maintained spontaneous breathing for ≥48 h with no need for any level of ventilator support after extubating, while 38 (43%) patients failed to wean. Clinical indicators, diaphragm ultrasound video, and diaphragmatic indicators (using automatic speckle-tracking measurement) were collected to construct the clinical/diaphragmatic dataset and diaphragm ultrasound dataset.

### Indicators Selection

Among the clinical or diaphragmatic indicators, each case contained 27 clinical and 6 diaphragmatic indicators (Table S1 of Multimedia Appendix 1). In this study, we selected height, weight, BMI, tidal volume, positive end-expiratory pressure, albumin, hemoglobin, age, temperature, systolic blood pressure, diastolic blood pressure, heart rate, respiratory rate, FiO2, PaO2, PaCO2, pH, serum sodium, serum potassium, white blood cell count, serum creatinine, serum calcium, serum magnesium, serum phosphorus, APACHE II score, SOFA (Sequential Organ Failure Assessment), and N-terminal pro-brain natriuretic peptide. In our previous retrospective study published in 2023 [14], clinical phenotypes of ventilated ICU patients to predict outcomes were derived from the eICU (electronic intensive care unit) Collaborative Research Database cohort and validated in the MIMIC-IV (Medical Information Mart for Intensive Care) cohort. Four clinical phenotypes were identified, which correlate with different clinical characteristics and outcomes, including 28-day mortality and extubation success. A total of 24 clinical variables were selected, including patient demographics, vital signs, blood gas analysis, laboratory measurements, scores, and MV parameters for phenotyping. Further, 18 of 24 variables in the abovementioned study were collected in the present cohort. Demographics were collected as baseline data upon ICU admission. Arterial blood gas analysis was performed at the end of the SBT. Ventilator parameters were recorded during the ultrasound examination. Additionally, a systematic review identified 21 factors associated with increased risk for reintubation, including higher APACHE II scores, low hemoglobin, low albumin, and high brain natriuretic peptide [15]. A case was reported in 2022 of iatrogenic hypermagnesemia, which presented as respiratory depression, preventing weaning from MV following cardiac surgery [16]. Hypophosphatemia was associated with respiratory muscular weakness, leading to weaning failure [17,18]. SOFA score during the first 24 hours of ICU admission represented significant independent predictors of diaphragm dysfunction and thus led to difficulties weaning from MV [19]. Laboratory markers, APACHE II, and SOFA scores were collected within 24 h before extubation. Furthermore, 6 diaphragmatic indicators were calculated using automatic speckle-tracking measurements based on the diaphragm ultrasound video. The detailed method and material were published previously [11].

### Data

For the diaphragm ultrasound videos, they were recorded with a high resolution of 900*1100 pixels and the length ranged from 48 to 556 frames. The percentage of the videos of 48‐150 frames is 30% and videos of 350‐556 frames is 30%. The overview of the patients and the details of the ultrasound video frame were presented in Table 1. During data preprocessing, the numeric variables and the categorical variables within the clinical indicators were preprocessed separately using 1-hot encoding, scaling, and normalization methods. The ultrasound videos were interpolated to a uniform size of 224*224*96. The size was unified considering the diaphragmatic excursion cycle and the model structure. The interpolation method was adapted from the bilinear interpolation algorithm in the Python (Python Software Foundation) package *SciPy*. Additionally, the data were normalized to a specific range of (0,1) before being encoded by the model.

**Table 1.** Overview of demographics, outcomes, and the characteristics of ultrasound video (N=88).

		Values
Age, mean (SD)		73 (14)
BMI, mean (SD)		22.40 (3.12)
Gender, n (%)		
	Male	60 (68)
	Female	28 (32)
Weaning outcome, n (%)		
	Success	50 (57)
	Failure	38 (43)
Ultrasound length (frame), n (%)		
	0‐50	8 (9)
	50‐150	17 (21)
	150‐250	16 (25)
	250‐350	28 (44)
	350‐450	13 (21)
	450‐560	6 (10)

### Multimodal Colearning Model

**Figure 1.**
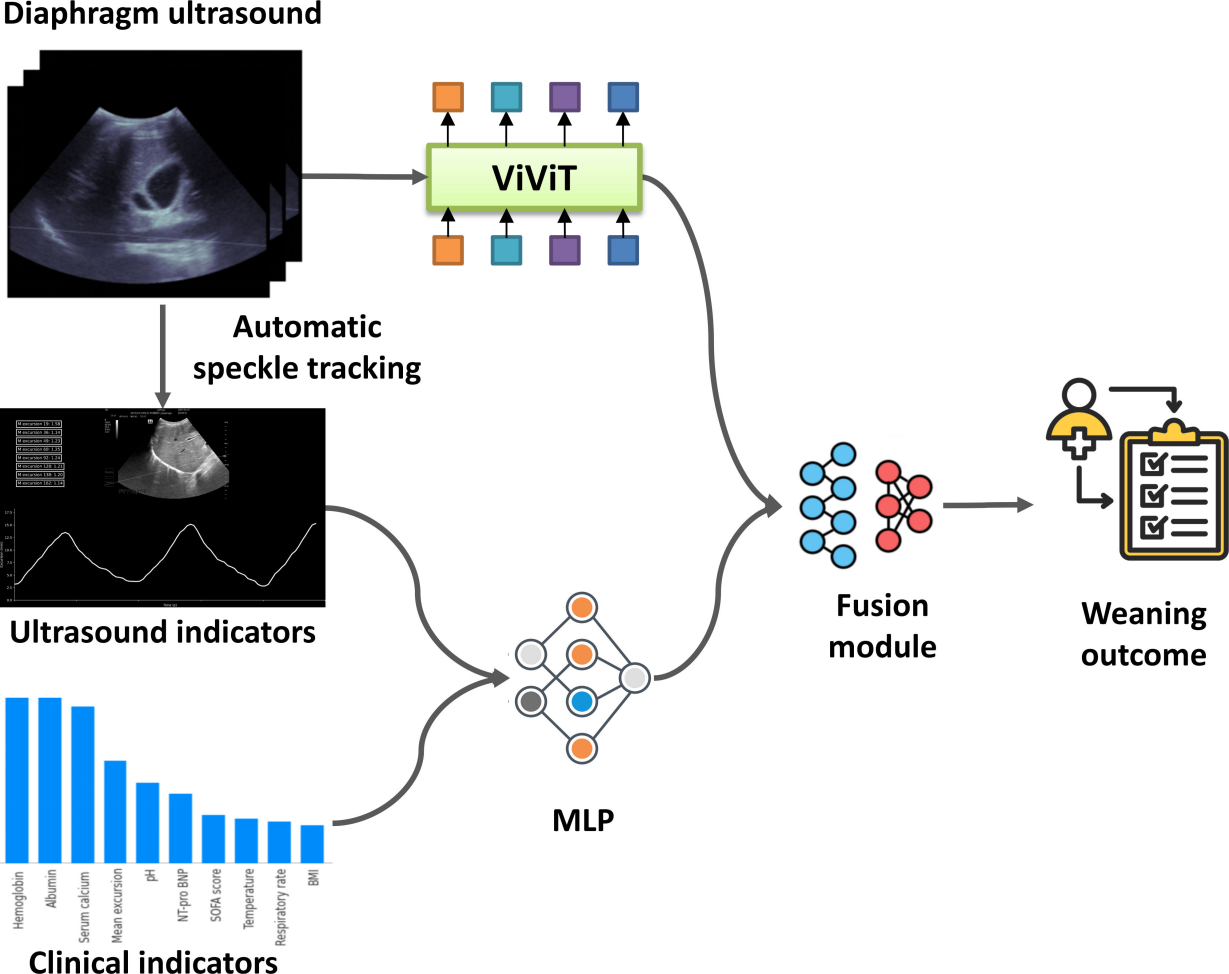
Overview of the proposed multimodal co-learning model. The model included 3 components: ViViT (ultrasound encoder), multilayer perceptron (indicator encoder), and Fusion module. MLP: Multilayer Perceptron; NT-proBNP: N-terminal pro–B-type natriuretic peptide; SOFA: Sequential Organ Failure Assessment; ViViT: Video Vision Transformer.

**Figure 2.**
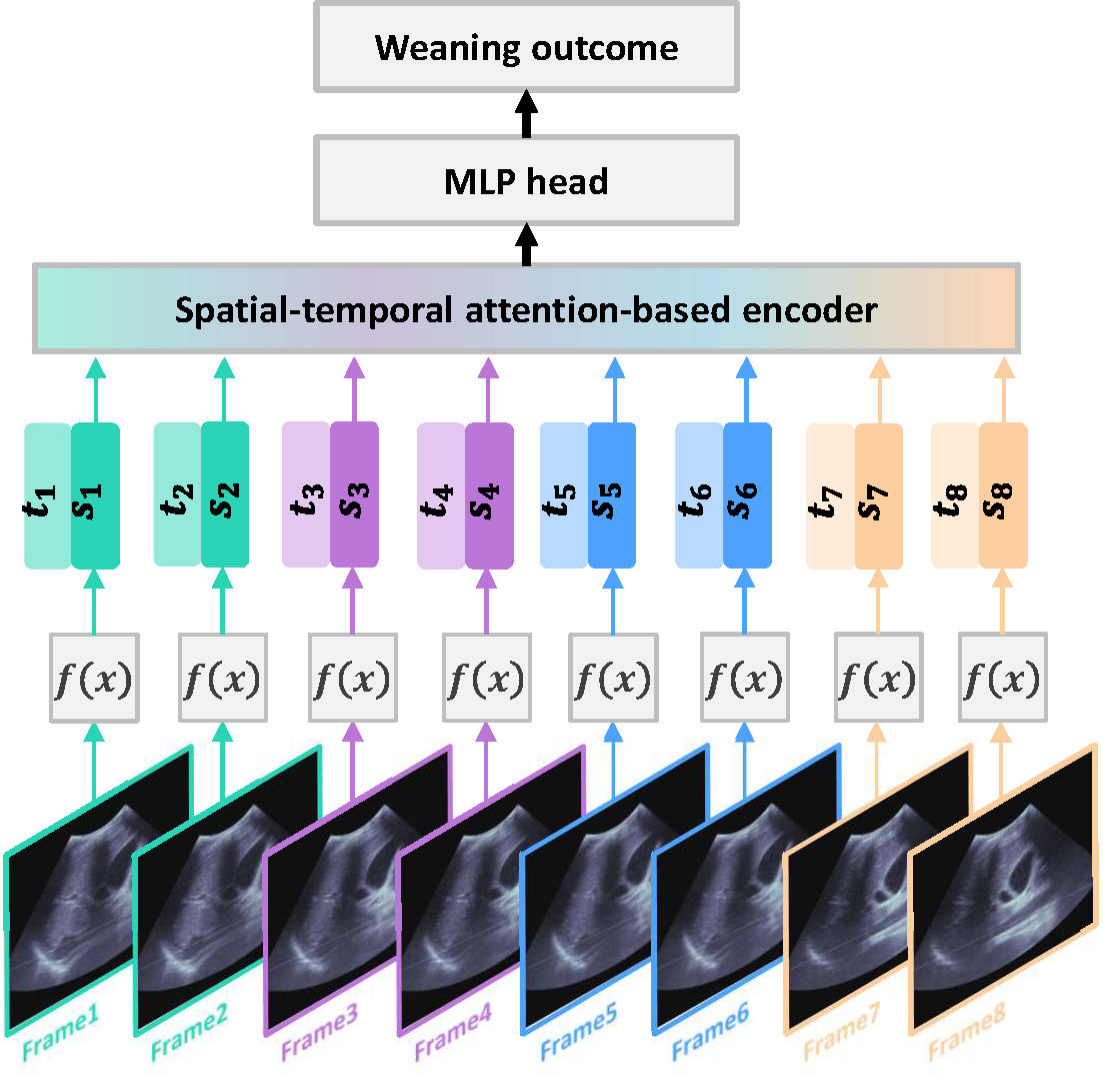
Structure of Video Vision Transformer. Ultrasound data were encoded into spatial feature ($s_{i}$) and temporal feature ($t_{i}$) and processed by a spatial-temporal encoder. MLP: Multilayer Perceptron.

A multimodal colearning model was then constructed (Figure 1), which simultaneously encoded both the video and clinical data and subsequently predicted weaning success from the integrated features. In this case, features would come from both the ultrasound videos (eg, image-based features) and the clinical indicators. The co-learning model had 3 components. First, Video Vision Transformer (ViViT) [20] was run as the ultrasound data encoder (Figure 2), aiming at extracting the ultrasound features. It included a spatial information encoder and a temporal information encoder based on the attention mechanism. Moreover, the ViViT captured temporal and spatial information at the same time, and for long videos, it also realized long-range sequence interaction through an attention mechanism. A small-scale grid search was used for pretraining. The learning rate for ViViT was between 1e-5 and 1e-4. The dropout rate was 0.5 to 0.7. We experimented with batch sizes of 4, 8, and 16 and set weight decay between 1e-4 and 1e-2. We trained the model for 50 to 100 epochs and applied an early stopping strategy (patience) with a range of 10 to 15 epochs.

Second, for the clinical and diaphragmatic indicators, a multilayer perceptron model, consisting of a sequence of fully connected layers and dropout layers, was adopted for clinical data encoding. Finally, in the fusion module, the ultrasound and clinical features were integrated. A multilayer perceptron classifier was used to obtain the final weaning outcome. In the multilayer perceptron classifier, 2 dropout layers were applied to avoid overfitting.

In the 5-fold cross-validation process, the 88 cases were randomly divided into training sets (n=60) and testing sets (n=18) in the ratio of 8:2. In each fold, 60 cases were randomly chosen as the training set, while the test set was the remaining 18 cases. In each fold, the model was trained on the training set and tested on the test set. The final result was calculated using the average results in the 5 folds. In the training process, the early stopping method was managed to avoid overfitting and improve model generalization performance. Receiver operating characteristic–area under the curve (AUC), precision-recall curve, and calibration curve for deep learning models were used to evaluate the results of the variant models. The model was developed and fit using Keras (Google LLC). The hyperparameters were optimized in the training process, with balanced class weights, binary cross-entropy loss, and 0.005 learning rate using the Adam optimizer.

Variable importance analysis was conducted to identify the key features influencing the prediction of weaning outcomes and to assess their relative impact. In deep learning models, currently, no universally accepted sensitivity analysis method fits all types of models. Therefore, we used the SHAP (Shapley Additive Explanation) method, a powerful technique for explaining individual predictions made by complex models. SHAP was applied to the classification of clinical indicators. Categorical features were processed using OneHotEncoder, and continuous features were normalized. SHAP values provide a measure of the contribution of each feature to a particular prediction, quantifying both the magnitude and direction (positive or negative) of their influence on the model’s output. SHAP values enabled a deeper understanding of the model’s behavior and the sensitivity of predictions to changes in individual indicators [21]. It should be noted that in this study, we incorporated SHAP to understand the importance of different features and enhance the interpretability, rather than to investigate causal relationships in clinical scenarios. SHAP plots generally do not directly show statistical significance (such as *P* values). To observe whether there is statistical significance, it needs to be verified through statistical tests. SHAP analysis can serve as a computer-aided diagnosis tool for clinicians, and its application requires integration with clinicians’ domain expertise to achieve better diagnostic outcomes.

### Ethical Considerations

All subjects’ families provided written informed consent. The study was performed following the approval of the ethics committee of Guangdong Provincial People’s Hospital (No. 2020-246H-1). All procedures were followed per the ethical standards of the responsible committee on human experimentation (institutional or regional) and with the Helsinki Declaration. Measures were taken to ensure participant privacy, including data anonymization and secure data storage. No financial or material compensation was provided to the participants or their families for participation in this study.

## Results

Four experiments were conducted on the prediction of weaning failure in an ablation setting, testing each component of the colearning model with corresponding data. To demonstrate the advantages of the proposed co-learning model in terms of modal interaction, the prediction performance was compared in a multimodal setting with 3 single-modal settings (diaphragm excursion, clinical or diaphragmatic indicators, and diaphragm ultrasound video). The 88 cases of ultrasound and clinical data were divided into training sets and test sets at a ratio of 8:2, and a 5-fold cross-validation was performed (Table 2). The results of the 4 experiments were demonstrated by classification accuracy, receiver operating characteristic curves, precision-recall curve, and calibration curve.

**Figure 3.**
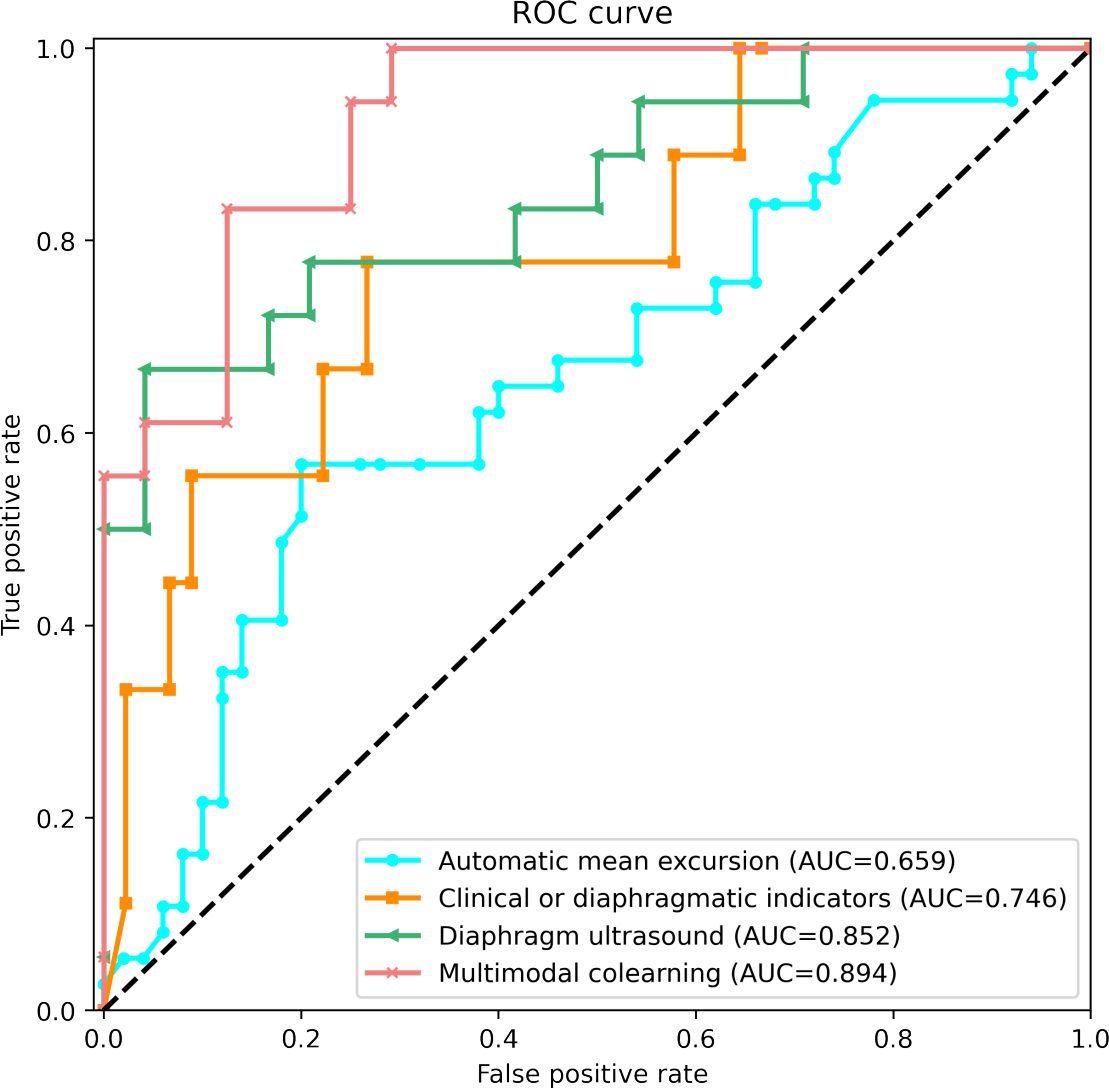
ROC curve of weaning failure prediction in 4 experiments. Pink curve: the multimodal co-learning (accuracy=0.8331, AUC=0.894), green curve: diaphragm ultrasound via ViViT (accuracy=0.8095, AUC=0.852), yellow curve: clinical or ultrasound indicators (accuracy=0.7381, AUC=0.746), and blue curve: automatic mean excursion via speckle-tracking measurement (AUC=0.659). AUC: area under curve; ROC: receiver operating characteristic; ViViT: Video Vision Transformer.

Initially, using automatic mean excursion as the sole predictor yielded a moderate performance with an AUC score of 0.659 [11], while the use of diaphragm ultrasound video data, processed with the ViViT model, resulted in an enhanced prediction performance, achieving a notable accuracy of 0.8095 and an AUC of 0.852. The prediction model based on a combination of clinical and ultrasound indicators provided a respectable performance (accuracy=0.7381, AUC=0.746). The most accurate and effective model was the multimodal co-learning approach. This model achieved an accuracy of 0.8331 and an AUC of 0.894, reflecting a significant improvement over the 3 single-modality approaches (Figure 3).

**Figure 4.**
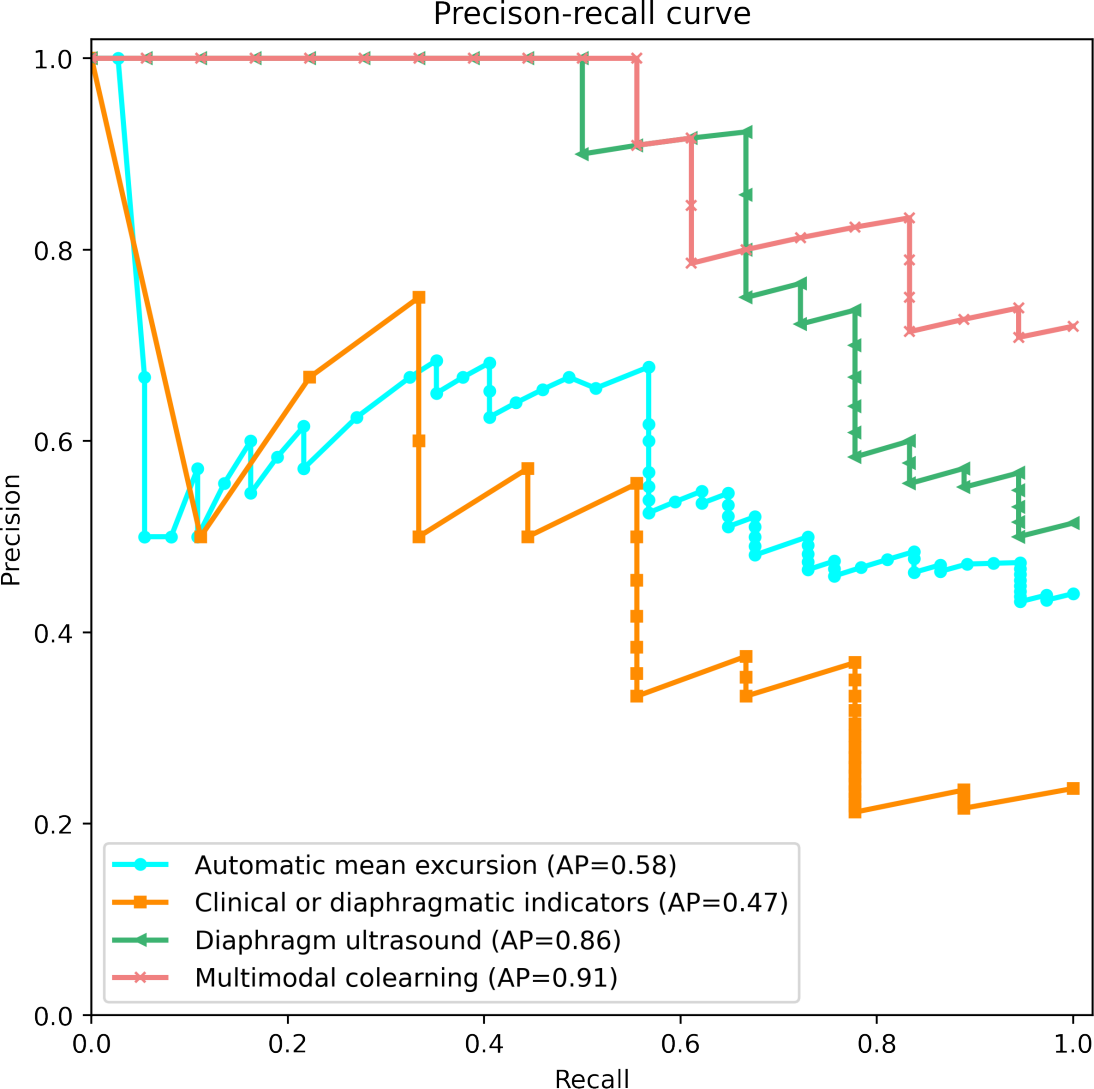
Precision-recall curve of weaning failure prediction in 4 experiments. Pink curve: the multimodal co-learning (AP=0.91), green curve: diaphragm ultrasound via ViViT (AP=0.86), yellow curve: clinical or ultrasound indicators (AP=0.47), and blue curve: automatic mean excursion via speckle-tracking measurement (AP=0.58). AP: average precision; ViViT: Video Vision Transformer.

Moreover, in the precision-recall curve, the average precision score was calculated to summarize a precision-recall curve as the weighted mean of precisions achieved at each threshold. The multimodal co-learning model ran the best compared to the other 3 single modalities. The proposed co-learning model achieved the highest score (average precision=0.91) among the 4 experiments (Figure 4).

**Figure 5.**
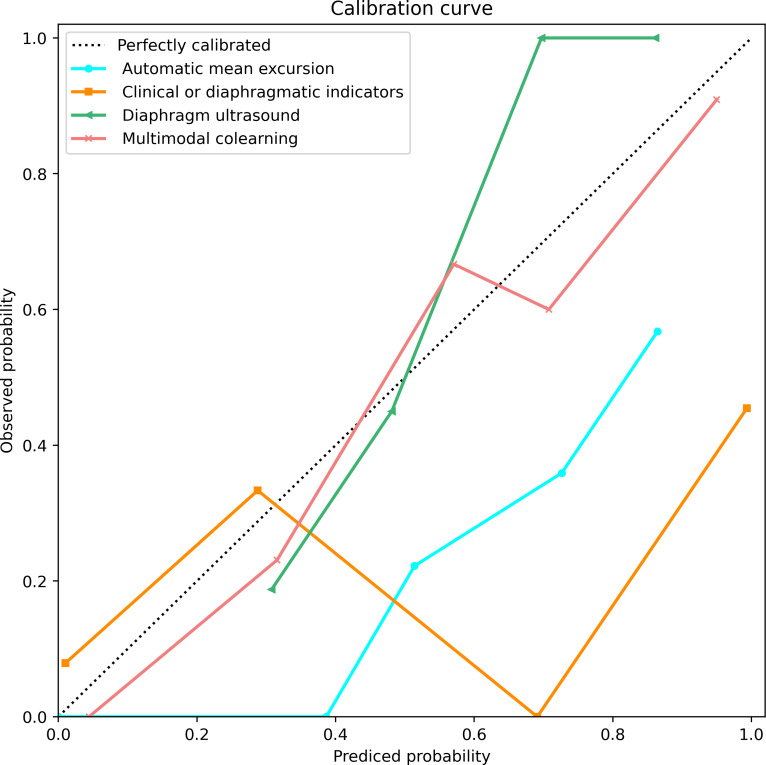
Calibration curve of weaning failure prediction in 4 experiments. Pink curve: the multimodal co-learning, green curve: diaphragm ultrasound via ViViT, yellow curve: clinical or ultrasound indicators, and blue curve: automatic mean excursion via speckle-tracking measurement. ViViT: Video Vision Transformer.

At last, the calibration curve was presented as a measurement of the confidence level of a proposed model and provided support for probability prediction (Figure 5). The proposed co-learning model was demonstrated to be well calibrated as the pink curve was closest to the perfectly calibrated line (the diagonal line at a 45-degree angle), showing that the predicted probabilities were highly consistent with the true outcomes.

**Table 2.** Results of 5-fold cross-validation.

Data	Colearning (AUC)^a^	ViViT^b^ (AUC)
1st-fold (n=18)	0.857	0.889
2nd-fold (n=18)	0.843	0.778
3rd-fold (n=18)	0.892	0.861
4th-fold (n=18)	0.924	0.843
5th-fold (n=18)	0.956	0.889

a AUC: area under curve.

b ViViT: Video Vision Transformer.

The experiments showed that the proposed co-learning model fully exploited the single-modal features and multimodal complementary features and achieved feature interaction between the modalities. Using both ultrasound and clinical data for colearning improved the accuracy of the weaning outcome prediction.

**Figure 6.**
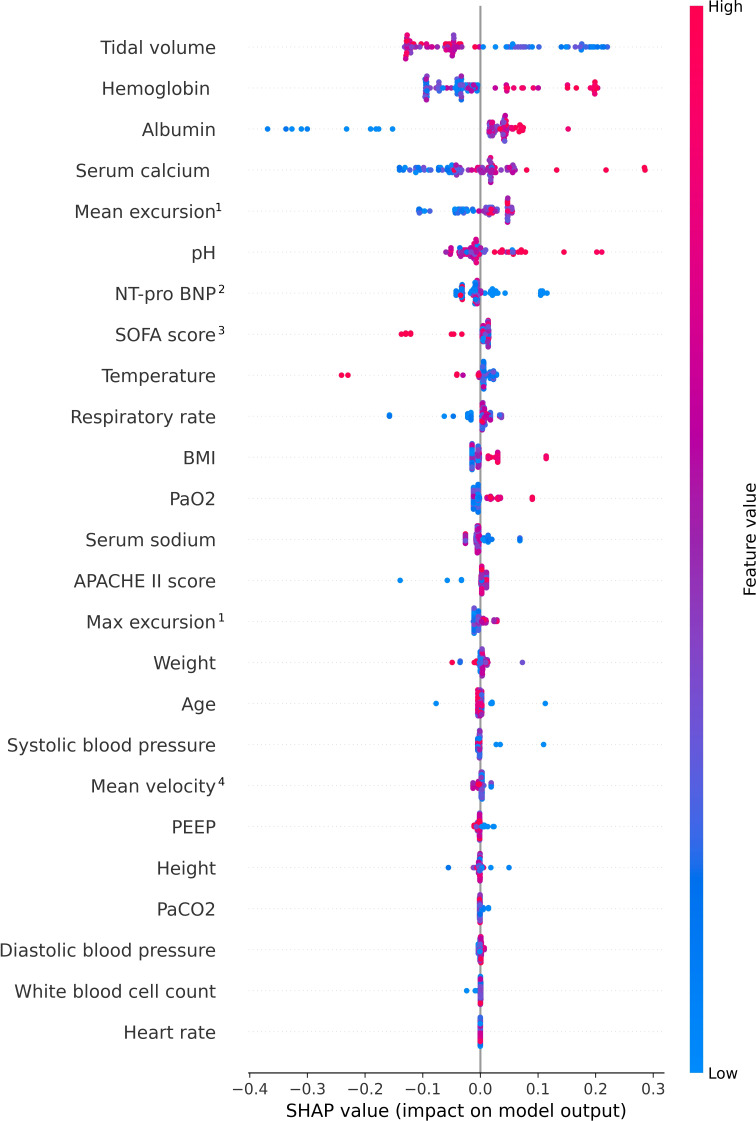
The SHAP values spot the impact on weaning outcome prediction. (1) Speckle-tracking automatic measurement of the diaphragm excursion, (2) N-terminal-pro-B-type natriuretic peptide, (3) SOFA score, and (4) speckle-tracking automatic measurement of the diaphragm excursion velocity. APACHE II: Acute Physiology and Chronic Health Evaluation II; PEEP: positive end-expiratory pressure; NT-pro BNP: N-terminal pro-brain natriuretic peptide; SHAP: Shapley Additive Explanation; SOFA: Sequential Organ Failure Assessment.

The SHAP values for the features were shown in Figure 6. The SHAP plot ranks features by their importance, with the highest importance at the top. The vertical axis ranked the features by the sum of the SHAP values across all samples, while the horizontal axis represented the SHAP value for each sample. Each dot corresponded to an individual sample, with the sample size indicated by vertical stacking of dots. On one hand, the color of each dot reflected the feature’s value, with redder colors corresponding to higher values and bluer colors to lower values. For example, tidal volume had the highest overall SHAP value. High tidal volumes (in red) clustered on the negative side of the SHAP axis (located to the left of the SHAP=0 line), suggesting that in this cohort, it negatively impacted weaning success. Furthermore, hemoglobin level was identified as a significant predictor, with higher hemoglobin values being associated with a greater likelihood of successful weaning. Albumin also emerged as an important factor in predicting weaning outcomes. The red dots were primarily concentrated on the right side of the SHAP=0 line, indicating that higher albumin levels were associated with successful weaning. However, lower albumin levels were linked to a higher probability of weaning failure, as evidenced by the blue dots located on the leftmost side of the albumin row. On the other hand, the importance of each feature was determined by the sum of its SHAP values, and the features with the highest sum of SHAP values were ranked at the top. Tidal volume, with the largest total SHAP value, emerged as the most influential feature for accurately predicting weaning outcomes. It should be noted that in this study, we incorporated SHAP to understand the importance of different features and enhance the interpretability, rather than to investigate causal relationships in clinical scenarios. SHAP plots generally do not directly show statistical significance (such as *P* values), which needs to be verified through statistical tests.

## Discussion

### The Clinical Challenge of Weaning and the Role of Diaphragm Dysfunction

About 26%‐39% of patients have trouble weaning [22,23] after resolution of the cause of respiratory failure and passing an SBT. Diaphragm dysfunction, resulting from weakness, atrophy, neurological damage, or mechanical disruption, is linked with prolonged MV duration, higher risk of weaning failure, longer length of ICU stay, and poor clinical outcomes [24]. Unsurprisingly, there is growing interest in investigating noninvasive bedside tools to evaluate the diaphragm and detect diaphragm dysfunction accurately. This is an important consideration when selecting which multiple parameters may be clinically useful to assess outcomes and for the prognostication of patients who may benefit from targeted interventions. Our serial research indicated that the incidence of diaphragm dysfunction in severe patients was as high as 75%, and diaphragm dysfunction was an important cause of weaning failure [25]. Over the past 10 years, diaphragm ultrasound has gradually become an extensively used tool for assessing diaphragm function [26]. As demonstrated in our previous studies, the ability to detect diaphragm dysfunction might become an important part of predicting weaning failure or prolonged weaning and holds the potential for determining weaning techniques. However, different studies have different predictors in addition to diaphragmatic indicators. Peer colleagues have also pointed out that the optimal prediction model has not been established, which is worth further exploration [27].

### Existing AI-Based Weaning Prediction Studies

This report discusses the potential of POCUS assessment of the diaphragm using automatic speckle-tracking measurement plus other clinical characteristics in predicting weaning outcomes. Integrated with diaphragmatic indicators and 27 clinical indexes, the multimodal model was constructed to achieve accurate and intelligent prediction of weaning outcomes (accuracy=0.8331, AUC=0.894). The results reflected a significant improvement over the other 3 single-modality approaches. The high AUC value indicated that the multimodal model had a strong ability to correctly classify the outcomes, suggesting that the fusion of diverse data types (clinical or ultrasound video) generated from the critical care scenarios allowed the model to capture a more comprehensive and complex set of patterns for better predictions. The use of AI algorithms or ML to build predictive models for the successful weaning process was promising. Derivation of different clinical phenotypes of ventilated patients from the MIMIC-IV cohort was associated with extubation success. A study conducted at Chi Mei Medical Center selected 26 feature variables to build the predictive models with AI/ML algorithms, with a high AUC ranging from 0.792 to 0.868 [28]. Cheng Hsin General Hospital collected 28 variables and created a simple AI model for predicting weaning time [29]. A retrospective cohort study in Central Taiwan showed that explainable ML could predict successful weaning among patients requiring prolonged MV (AUC=0.908) [30]. Moreover, the 2-stage AI prediction models effectively and precisely predicted the optimal timing to wean intubated patients, with the AUCs of optimal models ranging from 0.889 to 0.944 [13].

### Future Optimization 1: AI Integrates Multisystem POCUS Assessment

Of note, the result of this study was compromised due to a small sample. The AI model required a prospective cohort of a larger sample to validate its efficacy. Multiple risk factors were associated with weaning failure, such as cardiopulmonary dysfunction and older adults with severe comorbidities. It was urgent to optimize the decision-making process of weaning based on merely curtailing a few indicators. POCUS provides the dynamic assessment of multiorgan function during the weaning process. Even though high operator dependency limited the implementation and promotion of multisystem ultrasound monitoring in invasive MV patients, AI-integrated multisystem ultrasonic parameters rapidly and accurately assess, including for heart, lung, pleural cavity, diaphragm, and volume assessment.

### Future Optimization 2: Diaphragm-Protective Ventilation Strategy

For the concern raised by Sabourin et al [31], suggesting that diaphragm ultrasonography should be done in patients with spontaneous breathing without pressure support, we hope to refine the objective parameters such as laboratory indexes and MV settings to improve the prediction in a clinical scenario. We hypothesize that, with the assistance of AI, the multimodal model might potentially distinguish between MV support and the patient’s spontaneous breathing efforts, resembling clinical patterns seen in pressure support ventilation or synchronized intermittent mandatory ventilation mode. In our previous study, the excursion did not fully reflect whether the diaphragm movement was active or passive. Moreover, the value of diaphragmatic indicators was not only appreciated merely through excursion, but also in combination with the automatic measurement of diaphragm thickness and DTF. The abovementioned integration would become a strategy for diaphragm-protective ventilation.

### Limitations

First, an automatic measurement for DTF, a reliable indicator for the weaning outcome and supposedly less impacted by pressure support ventilation [31], was under development. Moreover, the success of the proposed model relied on the quality of diaphragm ultrasound videos. In clinical practice, variations in equipment quality and ICU doctors’ experience in performing POCUS could affect the reliability of these measurements. Future algorithms, integrating DTF and AI, would be explored to enhance accuracy and improve inter- and intraoperator reproducibility. Second, while the co-learning model achieved high predictive performance, the underlying decision-making process of the model was not fully interpretable. The translation of the findings of this study into clinical strategies still requires further refinement of the algorithm. Third, the relatively small sample size was not formally calculated, which limited the generalizability of the results to larger populations. A larger, multicenter study was needed to confirm the robustness and applicability of our findings across diverse patient cohorts. Recent literature, including 2 reviews published in 2022 [32] and 2024 [33], lacked evidence that combined ultrasound videos with AI for predicting weaning outcomes. As such, our proposed model from 2022 [20], integrating diaphragm ultrasound videos and AI techniques, represented its novelty in this specific context. In conclusion, the weaning outcome was closely related to multiple dynamic factors and the function of the diaphragm and even various organs. The integration of multimodal AI, using automatic measurement from POCUS and automated collection of objective clinical and laboratory indicators, greatly enhanced the prediction of weaning outcomes. This approach held promise for broader clinical application, a personalized tool that supported decision-making and was conducive to clinical promotion.

## Supplementary material

10.2196/72482 Multimedia Appendix 1 Table of clinical or diaphragm ultrasound indicators used in the model.

## Abbreviations

AI: artificial intelligence
AUC: area under the curve
DTF: diaphragm thickening fraction
eICU: electronic intensive care unit
ICU: intensive care unit
MIMIC-IV: Medical Information Mart for Intensive Care
ML: machine learning
MV: mechanical ventilation
POCUS: point-of-care ultrasonography
SBT: spontaneous breathing trial
SHAP: Shapley Additive Explanation
SOFA: Sequential Organ Failure Assessment
ViViT: Video Vision Transformer
