# Supplementary material for: AI Model Based on Diaphragm Ultrasound to Improve the Predictive Performance of Invasive Mechanical Ventilation Weaning: Prospective Cohort Study
Source: JMIR Form Res. 2025 Sep 8;9:e72482. doi: 10.2196/72482 (PMC12418127; doi:10.2196/72482)
Supplement: Multimedia Appendix 1 [file formative-v9-e72482-s001.docx]

**Table S1.** Clinical or diaphragm ultrasound indicators used in the model.

| indicators |
| --- |
| Height |
| Weight |
| Body mass index |
| Tidal volume |
| Positive end-expiratory pressure |
| Albumin |
| Hemoglobin |
| Age |
| Temperature |
| Systolic blood pressure |
| Diastolic blood pressure |
| Heart rate |
| Respiratory rate |
| FiO_2_ |
| paO_2_ |
| paCO_2_ |
| pH |
| Serum sodium |
| Serum potassium |
| White blood cell count |
| Serum creatinine |
| Serum calcium |
| Serum magnesium |
| Serum phosphorus |
| APACHE II score |
| SOFA |
| N-terminal pro-brain natriuretic peptide |
| mean excursion* |
| mean velocity* |
| maximal excursion* |
| maximal velocity* |
| minimal excursion* |
| minimal velocity* |

*Speckle-tracking automatic measurement of diaphragmatic movement [1].

Reference

**1.** Huang D, Song F, Luo B, et al. Using automatic speckle tracking imaging to measure diaphragm excursion and predict the outcome of mechanical ventilation weaning. *Crit Care.* Jan 14 2023;27(1):18.
